# The Reference Transcriptome of the Adult Female Biting Midge (*Culicoides sonorensis*) and Differential Gene Expression Profiling during Teneral, Blood, and Sucrose Feeding Conditions

**DOI:** 10.1371/journal.pone.0098123

**Published:** 2014-05-27

**Authors:** Dana Nayduch, Matthew B. Lee, Christopher A. Saski

**Affiliations:** 1 USDA-ARS, Arthropod-Borne Animal Diseases Research Unit, Center for Grain and Animal Health Research, Manhattan, Kansas, Unites States of America; 2 Clemson University Genomics Institute, Clemson University, Clemson, South Carolina, United States of America; Universidade Federal do Rio de Janeiro, Brazil

## Abstract

Unlike other important vectors such as mosquitoes and sandflies, genetic and genomic tools for *Culicoides* biting midges are lacking, despite the fact that they vector a large number of arboviruses and other pathogens impacting humans and domestic animals world-wide. In North America, female *Culicoides sonorensis* midges are important vectors of bluetongue virus (BTV) and epizootic hemorrhagic disease virus (EHDV), orbiviruses that cause significant disease in livestock and wildlife. Libraries of tissue-specific transcripts expressed in response to feeding and oral orbivirus challenge in *C. sonorensis* have previously been reported, but extensive genome-wide expression profiling in the midge has not. Here, we successfully used deep sequencing technologies to construct the first adult female *C. sonorensis* reference transcriptome, and utilized genome-wide expression profiling to elucidate the genetic response to blood and sucrose feeding over time. The adult female midge unigene consists of 19,041 genes, of which less than 7% are differentially expressed during the course of a sucrose meal, while up to 52% of the genes respond significantly in blood-fed midges, indicating hematophagy induces complex physiological processes. Many genes that were differentially expressed during blood feeding were associated with digestion (e.g. proteases, lipases), hematophagy (e.g., salivary proteins), and vitellogenesis, revealing many major metabolic and biological factors underlying these critical processes. Additionally, key genes in the vitellogenesis pathway were identified, which provides the first glimpse into the molecular basis of anautogeny for *C. sonorensis*. This is the first extensive transcriptome for this genus, which will serve as a framework for future expression studies, RNAi, and provide a rich dataset contributing to the ultimate goal of informing a reference genome assembly and annotation. Moreover, this study will serve as a foundation for subsequent studies of genome-wide expression analyses during early orbivirus infection and dissecting the molecular mechanisms behind vector competence in midges.

## Introduction

*Culicoides* biting midges (Diptera: Ceratopogonidae) are of medical and veterinary importance due to their ability to vector a number of viruses, protozoans, and nematodes. In North America, *Culicoides sonorensis* is suspected to be a main vector of bluetongue virus [1], [2] and epizootic hemorrhagic disease virus [3], [4], orbiviruses which cause hemorrhagic diseases and economic loss in ruminants such as sheep and deer. Both male and female biting midges are able to sustain themselves through sucrose feeding, which is usually acquired in the form of extrafloral nectar and serves as a quick energy source for flight [5]. However, females are anautogenous, and therefore rely on hematophagy in order to acquire nutritional resources necessary for egg development (vitellogenesis). Early morphological studies on various *Culicoides* species have estimated blood digestion to fully occur within 42–48 hours [6], and the commencement of vitellogenic processes can be seen as early as 24 h post-blood feeding.

Recent studies in other insect vectors such as sandflies and mosquitoes have provided insights in the temporal gene expression profiles associated with blood and sucrose meals [7], [8] and have illuminated dramatic changes within metabolic, digestive, immune, and reproduction signaling pathways across feeding states. The amount of sequence information pertaining to midges has been limited in comparison to these model organisms. Prior to the study here, there exists only a few *Culicoides* reports on tissue-specific transcript expression (e.g., EST libraries and qRTPCR) in response to feeding and oral orbivirus challenge [9], [10], which is an ultimate compromise in non-model genetic systems. Thus, the molecular functioning of this important vector only has been minimally explored.

The rapid evolution of next generation sequencing (NGS) has influenced a fundamental shift in genomic science where RNA sequencing technology (RNAseq) can provide a comprehensive picture of multiple steady-state transcriptomes and digital measure of gene expression in a single experiment [11]. This rapidly evolving technology affords scientists unparalleled opportunities to explore the transcriptome of practically any species in multiple ways such as comparative genomics [12], development of genotyping markers [13], and digital gene expression [14], [15]. In this study, we constructed and analyzed the first *de novo* transcriptome for female *Culicoides sonorensis* during non-feeding (teneral state), blood feeding (2–12 h after initial blood meal and 36 hours post-feeding), and sucrose feeding (2–12 h after initial sucrose meal and 36 hours post-feeding) using the Illumina HiSeq2000 platform. The objectives of this study were to perform a comprehensive comparison of digital gene expression profiles during these different feeding conditions, and to identify transcripts that may be relevant to key biological processes such as digestion, growth, and reproduction. The results of this study may be useful to further elucidating how midges function on a cellular and molecular level from a whole transcriptome perspective (possibly informing control strategies), and provide a rich data set to augment the ongoing genome-sequencing project [16].

## Materials and Methods

### *Culicoides sonorensis* feeding and RNA extraction

*Culicoides sonorensis* (Wirth & Jones) biting midges are maintained at the USDA-ARS Arthropod-Borne Animal Disease Research Unit. The AK colony used in this study was initiated from a field isolate from Owyhee Co., Idaho, August 1973 [17]. Female pupae collected from the colony were allowed to emerge in a chamber maintained at 26°C, 70–80% relatively humidity, with a 12–12 hour light-dark photoperiod. One to two day-old unmated adult female midges (n = 10) were collected for the teneral transcriptome libraries. A subset were divided into two containers and fed 10% sucrose for 1.5 hours or commercially available defribrinated sheep blood (Colorado Serum Company, Denver, CO) for 1 hour via an artificial membrane, then maintained on water *ad libitum*. For the “early” response transcriptome libraries, midges were pooled (n = 5 per time point; 15 total) at 2, 6, 12 h post-feeding, and for the “late” response transcriptome libraries midges (n = 10) were pooled at 36 h post-feeding. RNA extractions were performed with the RNeasy mini kit (Qiagen) following the manufacturer's instructions and quality was checked with an Implen NanoPhotometer. Total RNA integrity was assessed and assigned an RNA integrity number (RIN) using a 2100 Bioanalyzer (Agilent). Two biological replicates of each feeding state were performed, resulting in the construction of 10 transcriptome libraries (two of each: teneral, early and late sucrose-fed, early and late blood-fed).

### Illumina library construction, sequencing, and preprocessing

Whole transcriptome sequencing libraries for each sample was constructed and individually indexed using the TruSeq RNA sample preparation v2.0 kit (Illumina). Sequence libraries were multiplexed and sequence data collected on 3 lanes of a HiSeq2000 (Illumina) at the Genome Sequencing and Analysis Facility (University of Austin, Texas) using a 2×101 bp, paired end read type. Raw sequence reads were demultiplexed on the instrument and reads greater than or equal to 36 bp were cleaned of adapter and low quality bases with the Trimmomatic software package [18].

### *De novo* transcriptome assembly, annotation, and clustering

After read preprocessing, sequence reads were normalized for unique kmers for use in a global transcriptome assembly with the normalize_by_kmer_coverage.pl script that accompanies the Trinity software package [19]. A *de novo* transcriptome assembly of the adult female midge was constructed by combining normalized sequence reads from all samples with the Trinity software package [19] using the default assembly parameters with the addition of the –jaccard_clip option set. After the base assembly, the unigene was screened for internal stop codons and unlikely coding sequences with the TransDecoder plugin of the Trinity software package [19], and the resulting unigenes were clustered with the cdHit software [20] with a 98% identity threshold. Unigene sequences were annotated by BlastX alignment to the non-redundant protein database and the *Aedes aegypti* and *Culex quinquefasciatus* gene annotations with a 1e^−05^ expectation value, and assigned gene ontology terms (GO) [21]. Additionally, GO terms were slimmed using the GO-Slim function in Blast2GO v2.6.5 [22]. Potential chimeric contigs were inspected manually and removed when BLAST results suggested the fusion of two or more completely different reading frames. Unigene sequences were assigned to the functional groups biological process, molecular function, and cellular component according to the Gene Ontology hierarchy [23] with the AgBase functional classification tool [24], [25], [26]. This Transcriptome Shotgun Assembly project has been deposited at DDBJ/EMBL/GenBank under the accession GAWM00000000 and bioproject 238338. The version described in this paper is the first version, GAWM01000000; Short read sequences have been deposited to the NIH short read archive under the SRR Accessions: SRR1174028-SRR1174038; the 19,041 unigene assemblies can be found in GenBank nucleotide database under the following accessions: GAWM01000001- GAWM01019041.

### Differential gene expression profiling during teneral, blood, and sucrose feeding conditions

Comparative analysis of differential transcriptome responses between non-feeding (teneral), early blood meal (early blood), late blood meal (late blood), early sucrose meal (early sucrose), and late sucrose meal (late sucrose) to the *Culicoides* unigene was performed using the Tuxedo software package [27], [28], where reads were mapped to the unigene assembly with the Bowtie2 software [29]. Cufflinks was used to generate a transcriptome assembly for each condition and replicate, and Cuffmerge was used to merge transcriptome assemblies into one file for statistical analysis by the Cuffdiff software to identify genes whose expression profiles were statistically increased or decreased in abundance across the various feeding conditions over time. Differentially expressed genes were categorized into functional groups with the Agbase functional classification tool [24], [25], [26] to observe trends in molecular response to the different feeding conditions. Volcano and heat map plots were prepared using the R libraries, CummeRbund [30], and ggplot2 (www.ggplo2.org), respectively. In order to assess the presence of complete protein sequences of selected unigenes (e.g., those involved in vitellogenesis), manual annotation of translated queries was performed using InterproScan to search for functional domains and motifs (http://www.ebi.ac.uk/Tools/pfa/iprscan/).

## Results and Discussion

### RNAseq and transcriptome assembly

**Figure 1:**
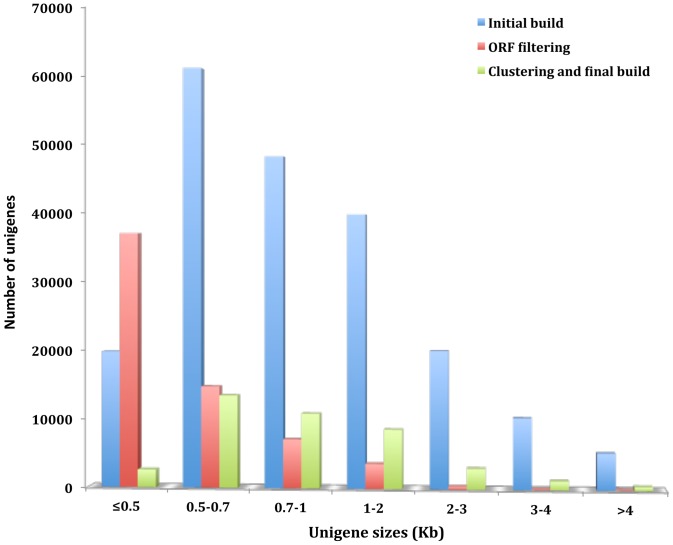
*Culicoides sonorensis* unigene assembly and filtering. Blue bars indicate the number of unigene sequences versus size that were present at the initial Trinity build. Red bars indicate the number and lengths of unigenes after ORF filtering, and green bars represent the final unigene build after clustering and manual inspection.

As improvements to next generation sequencing (NGS) technologies continue to be realized both in cost and data quality at an astounding pace, scientists are now afforded unprecedented opportunities to measure gene content and expression dynamics under many different conditions from multiple tissue sources in the same experimental design [31]. For non-model vector species having no available genome sequence, it is now routine to utilize NGS technologies to *de novo* sequence a relatively complete transcriptome and measure differential gene expression profiles to identify key biological pathways directed at dissecting molecular function. Applying these approaches in the midge can hopefully lead to the discovery of intervention points including system-specific biological control mechanisms [32], [33], [34]. Here, we deeply sequenced the transcriptome of *Culicoides sonorensis* under various feeding conditions that included non-feeding (teneral), blood feeding, and sucrose feeding on 3, 2×101 bp paired-end (PE) lanes using the Illumina HiSeq2000. Sequencing of the 3 lanes produced a total of 257.2 million pairs (average 25.7 million pairs per sample) after trimming with a length of 101 bp per sequence read, resulting in a total of 52 giga bases of transcriptome sequence (Table S1 in File S1). A *de novo* transcriptome assembly was performed using all reads for each condition and replicate with the Trinity software package [19] to produce a reference unigene set that encompassed the complete set of genes expressed under the experimental conditions in whole female midges. The transcriptome assembly consisted of 81,027 initial contigs as output from Trinity [35] with sizes ranging from 306 bp to 20 kb with a mean contig length of 1.5 kb (Fig. 1). The intractable complexities surrounding assemblies produced from short sequence reads alone, such as chimeric contigs, incomplete gene assemblies, and resolution of paralogous sequences, have significantly improved over the last few years as read lengths have increased nearly 3 fold, paired-end approaches increase mapping accuracies, and new sequencing chemistries are now available that are aimed to improve accuracies and reduce sequencing biases. However, it is still common for *de novo* transcriptome assemblies that are solely reconstructed from short-read sequences alone to consist of misassemblies, chimeric contigs, and artificial contigs as a result of inherent sequencing errors, genomic repeats, misassembly of homologous or paralogous genes, and artificial chimeric reads [36]. In our efforts to reduce redundancy and filter problematic or chimeric unigenes, we first screened the initial contig assembly for likely open reading frames (ORFs) where the longest ORF is identified within the contig and are scored according to the Markov model in each of the possible reading frames using the TransDecoder supplement of the Trinity software [35], which is largely based on the GeneID software [37] to remove contigs with internal stop codons and unlikely genuine coding sequences (Fig. 1). After ORF filtering (∼36% reduction in contigs), the 51,857 potential transcripts were clustered and filtered for redundancy using the cdHit-EST software (version 4.5.4) [20] that resulted in 20,183 unigenes, where the longest sequence out of a cluster of sequences that shared 98% sequence similarity was retained. A significant fraction of the collapsed contigs is assumed to be a result of allelic variance from pooling multiple individuals. A final round of manual inspection to remove chimeric contigs and resulted in a final reference set of 19,041 unigene sequences (Unigene Accessions: GAWM01000001- GAWM01019041) ranging from 300 bp to 7.8 kb with a mean transcript size of 1.3 kb (Fig. 1).

### Annotation and functional classification of the adult female *Culicoides sonorensis* transcriptome

**Figure 2:**
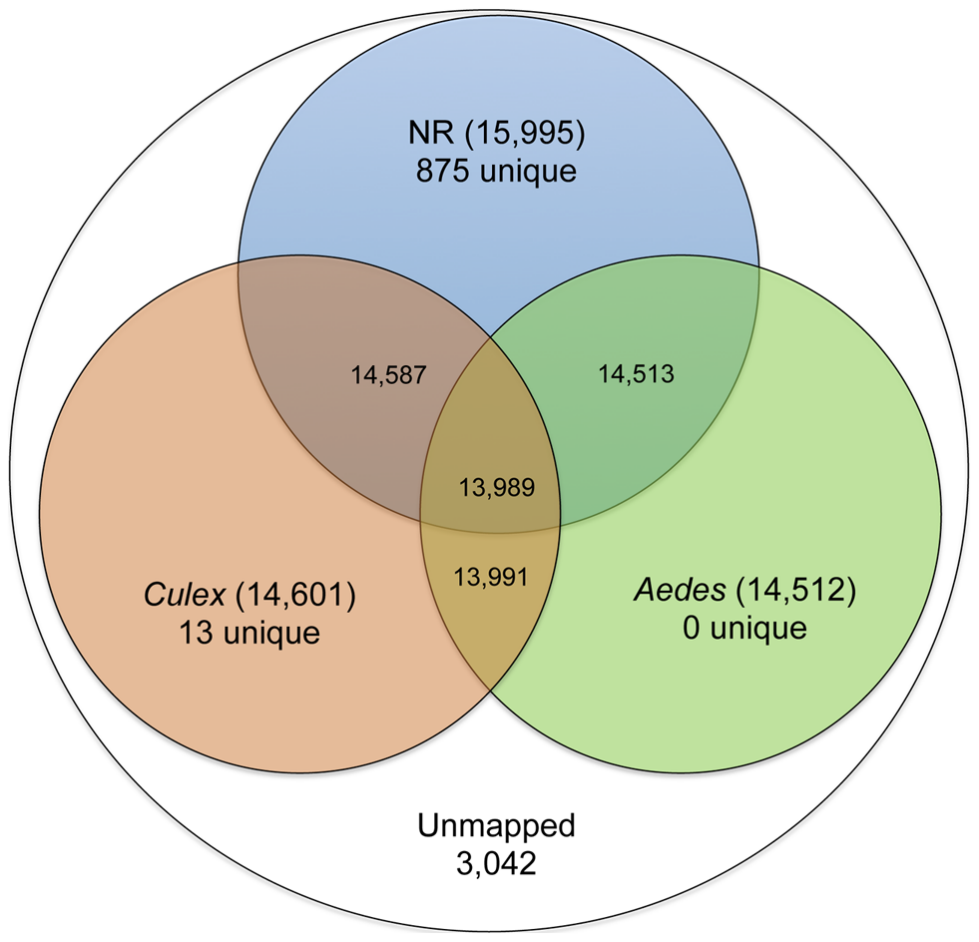
Unigene BLASTx alignment to the nr protein database, and *Aedes* or *Culex* gene set. The number of translated *Culicoides sonorensis* unigene alignments that have matches to the databases are shown. Unique numbers show how many *Culicoides* matches were unique to each of the databases, and the unmapped genes had not homology matches in any of the databases.

**Figure 3:**
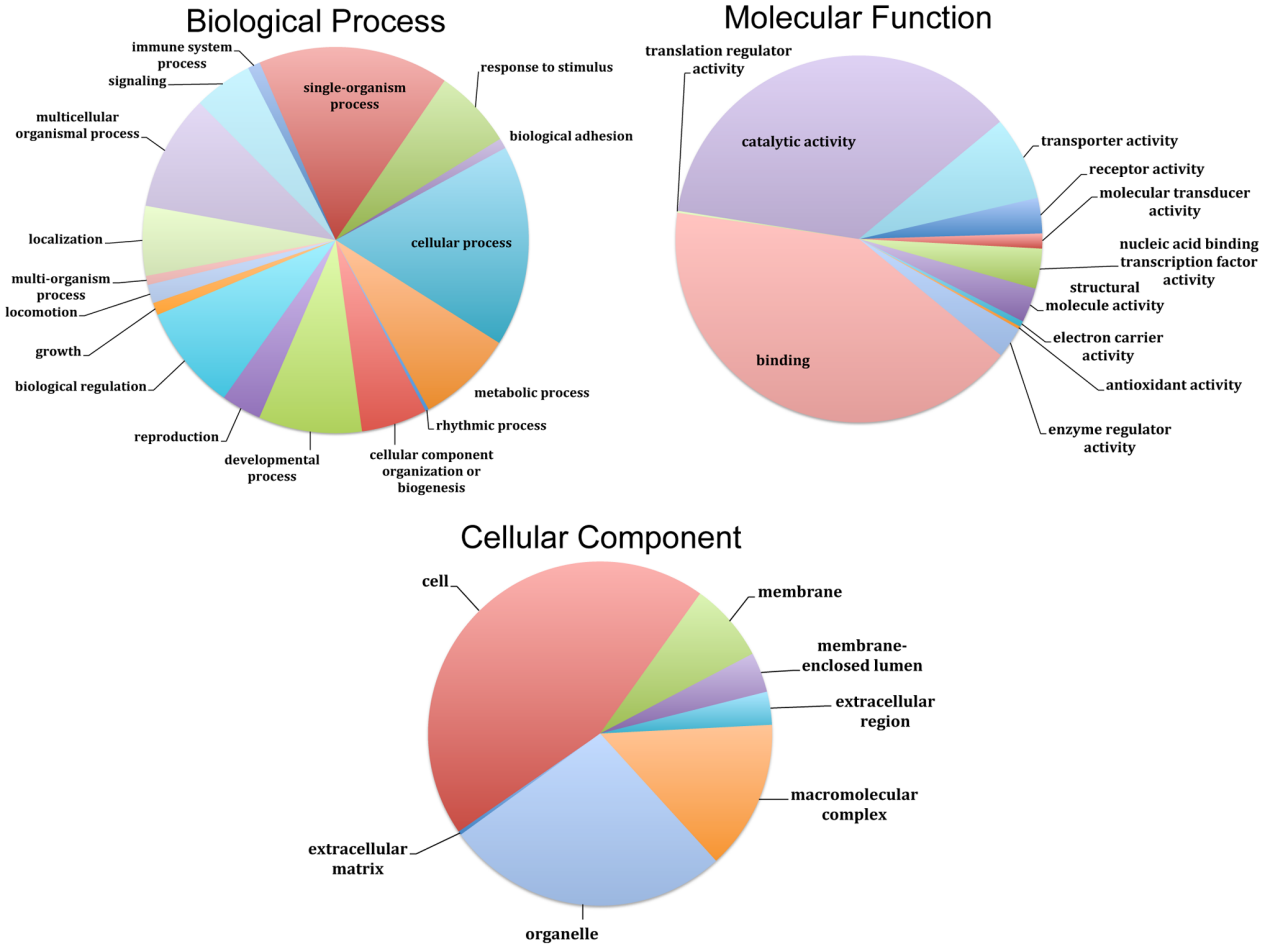
GO classification of the adult female midge unigene set. Classification and functional distrubuion of the 19,041 unigenes of the *C. sonorensis* transcriptome according to the 3 major classifications of the Gene Ontology: Biological Process, Molecular Function, and Cellular Component.

To annotate and determine potential functional roles, the complete unigene set was BlastX aligned to the closest well-annotated taxonomic relatives (*Aedes aegypti* and *Culex quinquefasciatus*) and the non-redundant (NR) peptide database at NCBI (e-value cutoff of ≤1e^−5^). This resulted in 14,512 (76%), 14,601 (76%), and 15,995 (84%) homologous sequences in *Aedes, Culex*, and NR, respectively (Fig. 2, Table S2 in File S1), suggesting a fairly comprehensive snapshot of the adult female transcriptome during the various feeding conditions. There were 3,042 unigenes that showed no homology to sequences deposited in these databases and may be unique to *Culicoides* (Fig. 2, Table S2 in File S1). Of these 3,042 unigenes, a significant fraction was differentially expressed during the various feeding conditions tested (detailed below), and is a priority list of genes for follow-up functional investigation which may lead to better understanding of the molecular functioning of the midge during these conditions. Gene Ontology (GO) terms were assigned to the 13,590 unigenes within the set using the Blast2GO software V.2.6.5 [38], which resulted in a total of 72,028 GO terms that ranged from levels 2 to level 6 and included both parent and child terms. We observed 14,139 accessions associated with cellular process, 36,758 associated with biological process, and 21,131 associated with molecular function (Table S3 in File S1). The 13,590 unigenes (71% of the total unigene) with GO terms were further categorized and classified according to the generic GO Slim categories (http://www.geneontology.org/GO.slims.shtml) using the Blast2GO software [39] to depict the female *Culicoides* functional profile during the different feeding conditions (both diet and temporally; Fig. 3). After broadly classifying the 19,041 genes that comprise the adult female *Culicoides* transcriptome, we identified an impressive distribution of categories containing genes and gene families associated with key functions such as metabolism, catalytic activity, development, immune system process, reproduction, binding, and response to stimulus (Fig. 3). Since *Culicoides* females are anautogenous, requiring a blood meal in order to produce eggs, a key aim of this study was to determine the genetic response during the course of a blood meal to reveal critical elements of the midge reproductive cycle and a candidate list of potential targets for validation and possible intervention strategy design. As a measure of completeness of biological capture in our feeding experimental design, genes categorized in Fig. 3 are representative of a large compliment of proteases involved in blood digestion, salivary gland proteins involved in hematophagy, vitellogenesis pathway intermediates (detailed below); we also identified genetic components of antimicrobial processes, which may be involved in vector competence.

### Transcriptome profiling during blood and sucrose feeding in *C. sonorensis* females

**Figure 4:**
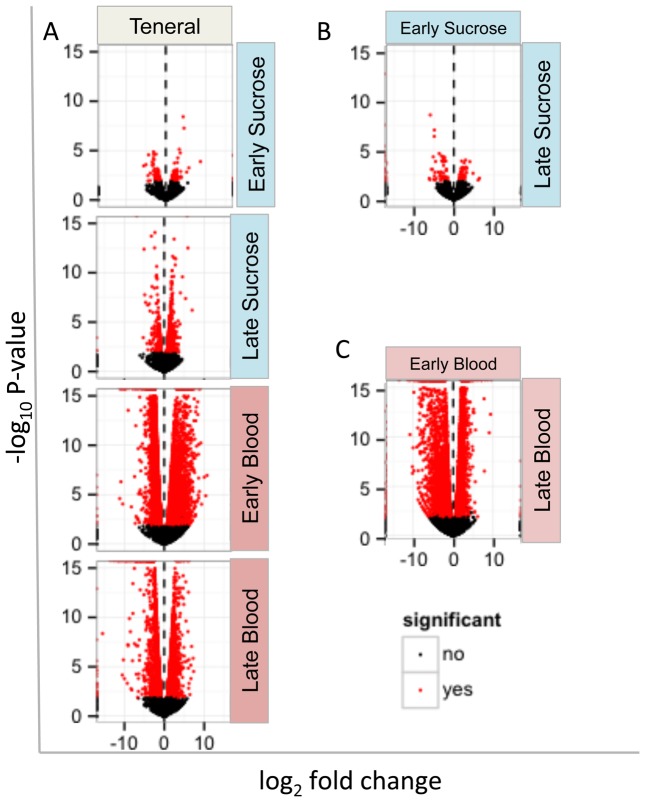
Volcano plots of pairwise comparisons illustrating diet effects on differential gene expression in *C. sonorensis*. The volcano plot is composed of comparisons of replicate normalized transcriptional states between feeding conditions including teneral, early (2, 6, 12 h pooled) or late (36 h) blood or sucrose feeding. Log2 fold change for each unigene (dots) is shown; red dots indicate statistical significance (P≤0.01) of the pairwise comparison of net transcript abundance (up or down to right or left of midline, respectively).

**Figure 5:**
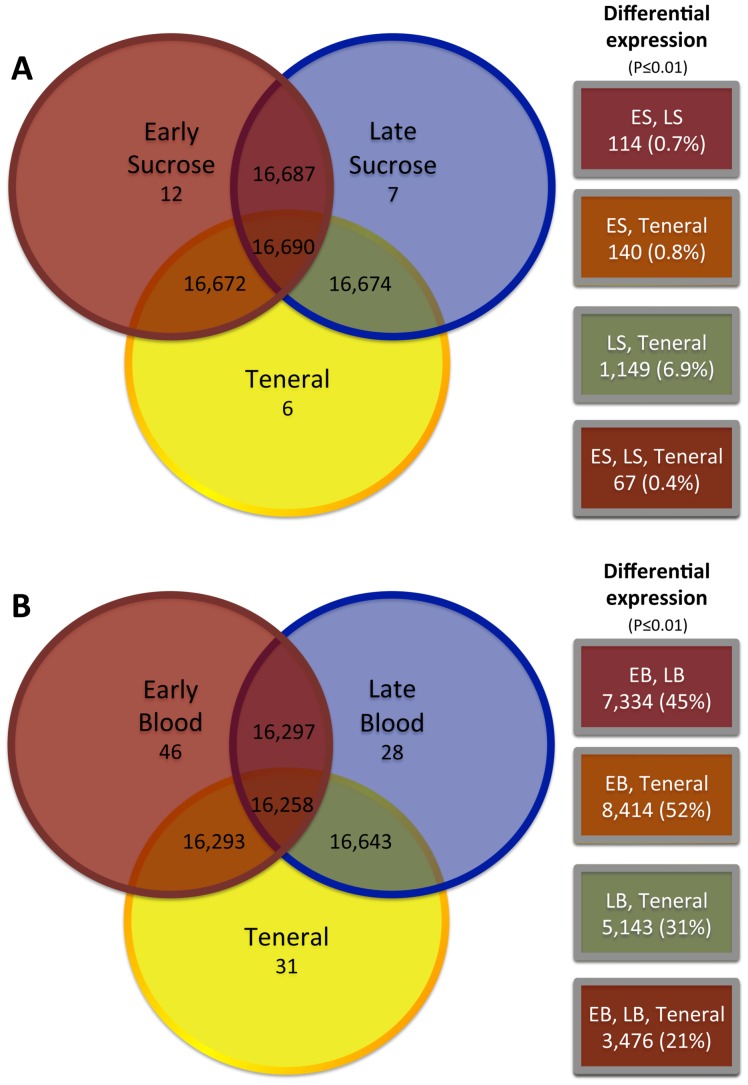
Transcriptome profiles and temporal expression analysis (early or late) within diet (sucrose or blood) in female *Culicoides sonorensis*. Comparison of the genes that were unique and shared between: A. teneral, early sucrose meal (2, 6, 12 h pooled; ES), and late sucrose meal (36 h; LS), or B. teneral, early blood meal (2, 6, 12 h pooled; EB), and late blood meal (36 h; LB). Right (boxes): The number of genes with differential expression profiles (P≤0.01), and the percent of shared unigene (in parentheses).

Determining the gene expression patterns in response to feeding over time is critical to our understanding of the molecular mechanisms behind development, vitellogenesis, and digestion. In our comparisons of sucrose and blood feeding over time to each other and the teneral state, we observed the greatest physiological response during blood feeding, when compared to teneral or sucrose feeding (Fig. 4). For example, the volcano plot comparison between teneral and early blood transcriptomes revealed 8,414 genes with significant magnitudes of change (red dots). Of these genes with significant fold change, 1,933 genes had P-values between 0.001 and 0.01, 1,326 genes between 0.0001 and 0.001, and 4,857 genes with P-values less than 0.0001. Genes with the largest abundance fold change included lipase, serine protease, trypsin, vitellogenin, and novel *Culicoides* transcripts (upregulated), and glycine N-methyltransferase, salivary protease inhibitor, and mitotic protein phosphatase regulator (downregulated). In the teneral vs. late blood comparison, 1,766 genes had P-values between 0.001 and 0.01, 955 genes had P-values between 0.001 and 0.0001, and 2,123 genes with P-values less than 0.0001. Similarly, late blood genes with the greatest fold change in comparison to teneral include proteases, trypsins, vitellogenins, and unknown genes (upregulated), and glycoside hydrolases, odorant receptors, and novel genes (downregulated). Of particular interest, we observed a net gene upregulation (∼60%) of transcript abundance immediately after the blood meal (Fig. 4A, Teneral vs. Early Blood), and a net downregulation (∼56% of the 7,334) of transcript abundance at the late time point after the blood meal as is seen by the density of red dots in the negative scale (Fig. 4C, Early Blood vs. Late Blood). To elucidate the feeding response among diet source over time, we compared the genes responding between teneral feeding, early sucrose/blood meal, and late sucrose/blood meal (Fig. 4; Fig. 5). Comparison of sucrose feeding conditions over time with teneral (Fig. 5A) revealed relatively similar transcriptomes with only a handful of unique genes measurable among each transcriptome. Similarly, the number of genes that were differentially expressed across the sucrose diet over time was comparatively low, with the largest observed difference between the late sucrose feeding and teneral transcriptomes. The early and late sucrose transcriptomes were nearly identical, showing only a few genes with significantly different expression profiles (114 total); this included genes for digestive enzymes whose transcript abundance decreased over time. The minor differences in expression profiles suggest that the genetic landscape behind sucrose feeding is very similar to non-feeding conditions.

**Figure 6:**
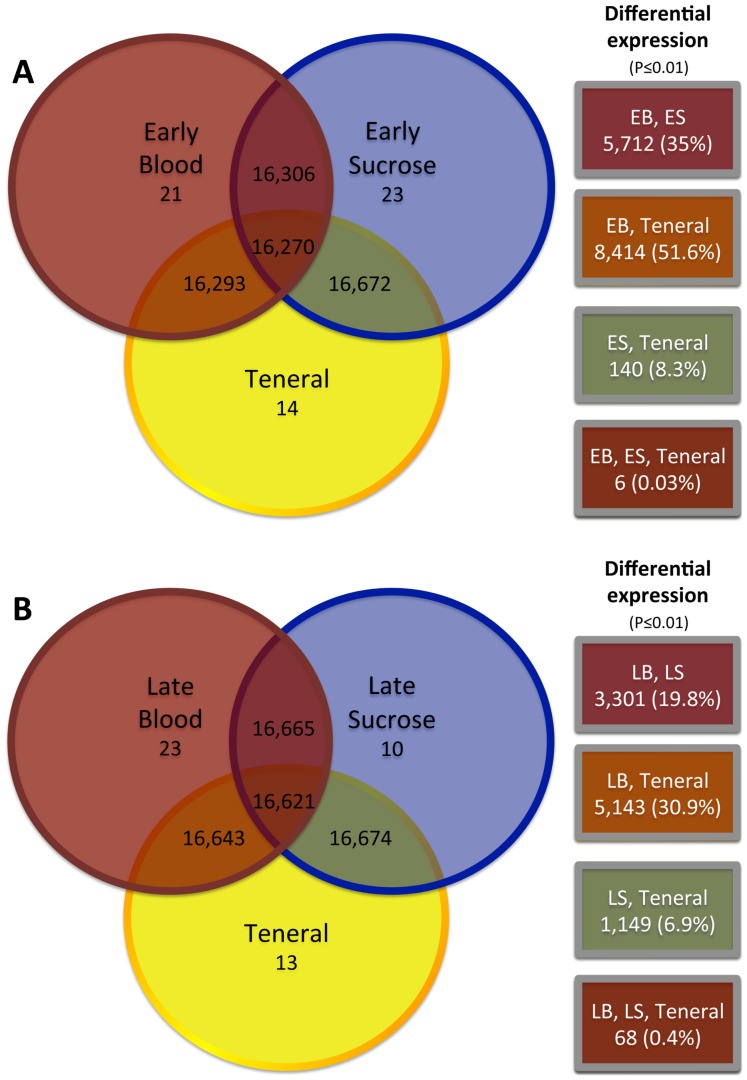
Transcriptome profiles and temporal expression analysis (early or late) across diet (sucrose or blood) in female *Culicoides sonorensis*. Comparison of the genes that were unique and shared between: A. teneral and early blood (EB) and sucrose (ES) meals (both 2, 6, 12 h post ingestion, pooled), or B. teneral and late blood (LB) and sucrose (LS) meals (both 36 h). Right (boxes): The number of genes with differential expression profiles (P≤0.01), and the percent of shared unigene (in parentheses).

Comparisons of the early and late blood transcriptomes with the teneral conditions (Fig. 5B) revealed a measurable genetic landscape with most of the same genes captured in each of the conditions, but the expression profiles differed noticeably between these conditions. For example, the most significant genetic response occurred during the 12 h interval after the blood meal, where we observed 8,414 genes with differential expression profiles between the two transcriptomes, nearly half of the genes shared between these two conditions. Similarly, 36 h after the course of the blood meal, the transcriptome still was responding significantly with 5,143 genes differentially expressed compared to teneral female midges (Fig. 5B). Comparisons between the early blood, late blood, teneral comparison had 3,476 overlapping genes with differential expression profiles (Fig. 5B) suggesting the physiological response to blood feeding was drastically different than the response to sucrose feeding, where the comparisons revealed only 67 differentially-expressed genes (Fig. 5A). We also compared the effects of diet within time, and similarly found that a number of genes overlapped between transcriptomes, but significant numbers of genes showed differential expression profiles across the blood but not the sucrose feeding conditions (Fig. 6). The most significant differences were observed between the early blood (EB) and early sucrose (ES) transcriptomes, where 5,712 genes, 35% of the total number shared, were differentially expressed (Fig. 6A). In contrast, fewer differences were seen between the late blood (LB) and late sucrose (LS) transcriptomes (3,300 genes, 19.8% of total number shared) (Fig. 6B). The comparison of the LB, LS and teneral transcriptomes showed only 68 differentially expressed genes, which implies that 36 h after feeding on either diet, a large part of the expression profile is returning to a condition similar to that seen in non-feeding midges (Fig. 6B).

**Figure 7:**
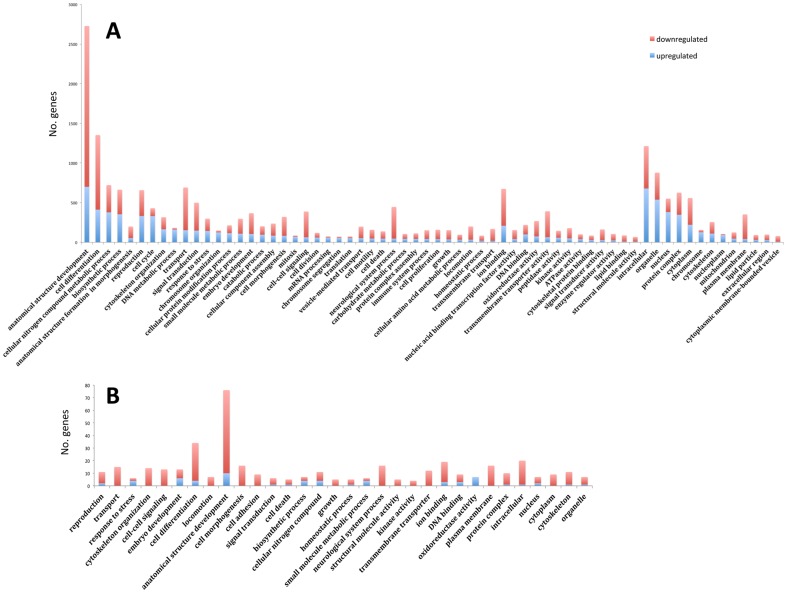
Global response to blood and sucrose meals in female *Culicoides sonorensis*. Global transcript upregulation (blue) and downregulation (red) in response to a blood meal (A; early and late transcriptomes combined) or sucrose meal (B; early and late transcriptomes combined). Note difference in vertical scale between A and B.

The genes with differential expression profiles identified during the course of both the blood and sucrose meals were categorized according to GO classifications to depict global differences between blood and sucrose feeding (Fig. 7). The physiological response to blood feeding was strikingly different from the teneral state, while sucrose feeding showed fewer differences in response; this stark contrast between the two diet sources was evident in both in the diversity of categories and the number of genes differentially expressed in those feeding conditions. For example, sucrose feeding caused differential expression of <20 genes in all but two of the categories (Fig. 7B). Conversely, the blood meal induced a much more complex physiological response that was observed in several categories, such as anatomical structure development, organelle, reproduction, cell cycle, and others, that involved hundreds to thousands of genes (Fig. 7A).

### *C. sonorensis* gene expression profiles in response to blood feeding gives insights into molecular basis of hematophagy

**Figure 8:**
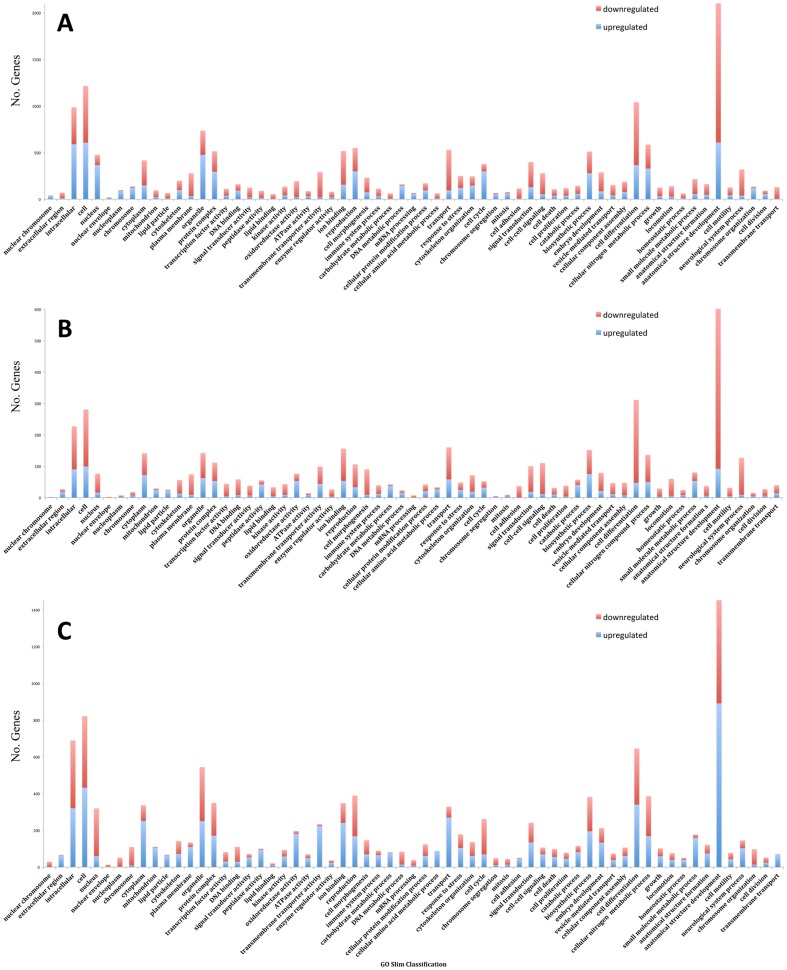
Transcriptional response to blood feeding in female *Culicoides sonorensis*. Numbers of genes classified within GO-slim categories that were upregulated (blue) or downregulated (red) in response to blood feeding are shown, including comparisons of (A) teneral versus an early blood meal (B) teneral versus an late blood meal, and (C) early blood meal versus late blood meal. Note difference in vertical scale between A, B and C.

Genes with differential expression profiles (at least 2-fold abundance increase or decrease, P<0.01) in response to blood feeding were further classified into functional categories in order to observe trends in molecular function (Fig. 8). In comparison to the teneral midges, the early blood fed midges showed the strongest genetic response through transcript abundance changes in genes classified within anatomical structure development, cellular differentiation, reproduction, signal transduction, response to stress, transport, embryo development, lipid and ion binding, and carbohydrate metabolism (Fig. 8A). Table S4 in File S1 is a list of the top 100 genes with at least a 2-fold increase or decrease in transcript abundance as a result of an early blood meal. Intriguingly, 36 of the top 100 induced genes had no homology to other organisms, revealing new targets for functional classification. Not surprisingly, many genes that were differentially expressed in early blood feeding (Table S5 and Table S6 in File S1) are associated with blood digestion (e.g. proteases, lipases), hematophagy (e.g., salivary proteins), and vitellogenesis. At 36 h post blood feeding, we observed a general decrease in the number of genes with differential expression profiles (Fig. 8B; Table S5 and Table S7 in File S1), suggesting that many major metabolic and biological events unfold within 12 h post blood meal and the expression of these early response genes subsides within the window of 36 h. Comparison of the early and late blood-fed midge transcriptomes revealed 7,334 differentially expressed genes (Fig. 5B; Table S8 and Table S5 in File S1) categorized within anatomical structure and cell differentiation, transport, ion binding, oxidoreductase, signal transduction, embryo development, response to stress, and peptidase activity, were more significantly upregulated in comparison to the measurement at early blood feeding, while categories such as reproduction, DNA binding, and cell cycle were downregulated (Fig. 8C; Table S5 in File S1). Of the genes differentially expressed during the blood meal over time (Fig. 8C), 787 had no homology hits to the databases, possibly revealing new *Culicoides*-specific functional targets for gene knockouts and evaluation. Genes that were highly downregulated in abundance between early and late blood-fed midges were lipases, takeout-like protein (involved in taste and olfactory sensing [40]), pyruvate metabolism, vitellogenins, and attacins suggesting these processes were either required or induced earlier in the blood metabolism process. Genes that were highly upregulated between the early and late blood-fed conditions including chitinases, trypsins, serine collagenases, tankyrase, other lipases, salivary proteins, and many other digestion related genes suggests they were utilized later in the blood digestion process.

In comparison to the blood feeding process, and in congruence with the stark contrasts seen in Figs. 5–7, the genetic response to sucrose feeding was unremarkable, where both the early sucrose and late sucrose transcriptomes were relatively similar to the non-feeding teneral state (Fig. 5A, Fig. S1). We observed only 140 differentially expressed genes right after a sucrose meal, and 1,149 at 36 hours post feeding, compared to teneral midges (Fig. 5, Fig. S1; Table S9 and Table S10 in File S1). Categories containing genes with altered expression profiles that responded to early sucrose feeding were anatomical structure development, embryo development, DNA binding, and response to stress (<1.5% of the blood induced profile) (Fig. S1, Table S11 in File S1). In the 36 h post-sucrose transcriptome, we observed a larger physiological response and a general trend in transcript abundance decrease in anatomical structure development, cell differentiation, ion binding, reproduction, and signal transduction categories when compared to teneral midges (Fig. S1; Table S11 in File S1), with the exception of a net abundance increase in the oxidoreductase superfamily, which has been previously described with salivary activity in insects [41], [42]. Comparison of sucrose feeding over time (Fig. S1), showed a decrease in the expression of ion binding genes, and a significant increase in genes classified as anatomical structure development, cellular nitrogen metabolic process, small molecule metabolic process, oxidoreductase activity, and transferase activity.

### Differential expression analysis sheds light on anautogeny and vitellogenesis in *C. sonorensis*

Like many but not all hematophagous vectors, female *Culicoides sonorensis* are anautogenous, requiring a blood meal in order to successfully provision nutrients to the developing oocytes and produce eggs (vitellogenesis). The processes underlying vitellogenesis have been well described in mosquitoes, and result in stimulation of the fat body to induce production of yolk protein precursors (YPPs) that will be transported to the ovary for incorporation in oocytes [43], [44]. YPPs include vitellogenin (Vg), a precursor of the yolk storage protein vitellin, lipophorin (Lp), a lipid transporting lipoprotein, vitellogenic carboxypeptidase (VCP) and vitellogenic cathepsin B (VCB). In female mosquitoes, the presence of a blood meal in the midgut causes the brain to produce and release hormones such as ovarian ecdysiotropic hormone (OEH) and insulin-like peptides (ILP), which subsequently stimulate the ovaries to produce ecdysone, and secrete it into the hemolymph [43], [44], [45]. Ecdysone is converted into 20-hydroxyecdysone (20E) in target tissues such as the fat body and subsequently binds a heterodimeric nuclear receptor, comprised of ecdysone receptor (EcR) and Ultraspiracle (USP). This complex translocates to the nucleus and binds EcR/USP response elements on DNA, activating transcription of early response genes (e.g., E74, E75) as well as YPPs [44]. Nutritional signals from the blood meal itself (e.g., amino acids) also work cooperatively with ILPs to co-stimulate YPP synthesis by the fat body via the TOR (target of rapamycin) pathway [46]. In mosquitoes, at around 36 h post-bloodmeal, the 20E levels drop, and the fat body resumes its function as a metabolic, nutrient storage and immune organ. The cyclicity of gonadotrophic cycles is controlled on a transcriptional level in mosquitoes by seven-up (*svp*), a gene whose product replaces EcR in heterodimers when 20E titer declines, and therefore prevents activation of YPP genes during developmental arrest in between blood meals [47]. This maintains the previtellogenic state of arrest although other regulatory mechanisms exist [44], [48].

**Figure 9:**
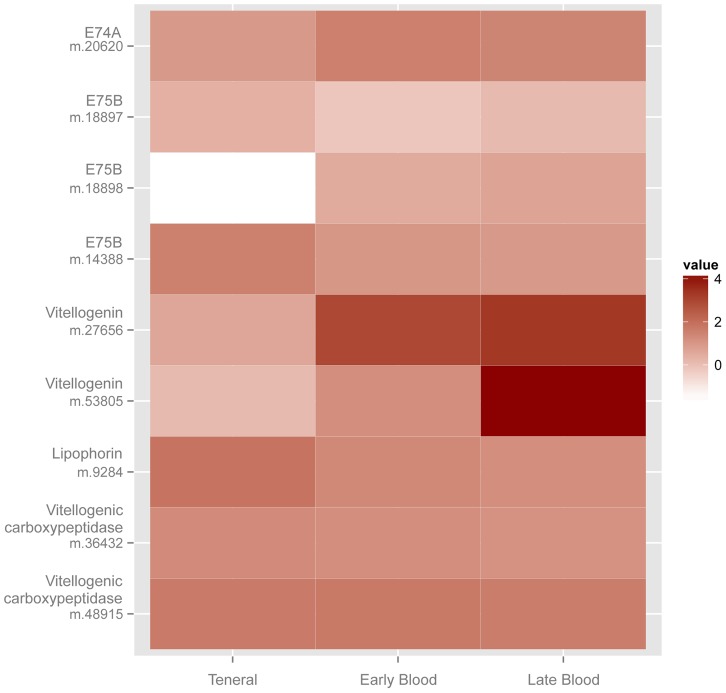
Heatmap showing comparative expression of *Culicoides sonorensis* vitellogenesis target genes in response to blood feeding. Early blood is 2, 6, 12(pooled) and late blood is 36 h post ingestion, with Log_10_ FPKM values indicated in legend. Further description of these genes can be found in Table 1, and detailed statistic and values of fold change can be found in Table 2.

**Figure 10:**
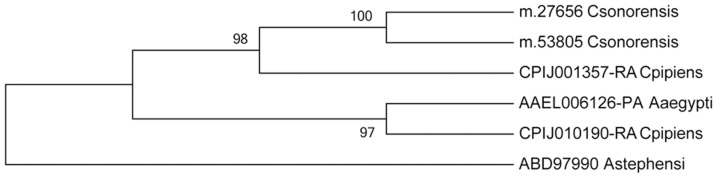
Maximum likelihood tree for *Culicoides sonorensis* and mosquito vitellogenins. Consensus tree for full protein sequence of vitellogenins is shown for *Culicoides sonorensis* (including unigene number) and mosquitoes (including Genbank accession numbers). The percentage of replicate trees in which the associated taxa clustered together in the bootstrap test (1000 replicates) are shown next to the branches Evolutionary analyses were conducted in Mega5 (www.megasoftware.net). ClustalW alignment used for generating this tree is available in Fig. S2.

Homologs for most of the components of this vitellogenic pathway were found in the *C. sonorensis* female transcriptome, and included peptide hormones, nuclear receptors, target genes, the seven-up regulator and target receptors (Table 1). Differential expression analysis of vitellogenic genes showed that blood feeding induced two insulin-like peptides, two USP nuclear receptors, both early response genes (E74A, E75B), both vitellogenins and vitellogenic carboxypeptidase; all were variably induced in either the late or early blood feeding transcriptomes (P≤0.01; Fig. 9, Table 2). The genes with the highest upregulation in response to blood feeding were the vitellogenins, with Vg m.27656 (Acc. no. GAWM01006233) showing the greatest upregulation in the early blood feeding transcriptome (8.03 Log_2_-fold change) and Vg m.53805 (Acc. no. GAWM01013694) being induced in the late blood-fed state (>14.04 Log_2_-fold change). ClustalW alignment and maximum likelihood phylogenetic analysis showed that the *C. sonorensis* vitellogenins were most similar to a vitellogenin from *Culex pipiens* (Fig. 10; Fig. S2), which is also induced after blood feeding in anautogenous mosquitoes [49]. The expression of several vitellogenesis-related genes differed over time in blood-fed midges (i.e. early vs. late blood-fed transcriptome comparisons, Table S8 in File S1; P≤0.01) and included: two insulin-like peptides (upregulated), two USP receptors (downregulated), early response genes (variably up or downregulated), vitellogenins (one downregulated, one upregulated), two vitellogenic carboxypeptidases (both upregulated), seven-up (all upregulated) and one lipophorin receptor (upregulated), which was similar to expression patterns seen after blood feeding in other vectors [43], [50], [51].

**Table 1:** Genetic components of vitellogenesis in *C. sonorensis*.

Categories and homologs	Acc. no.	Seq. no.	*Aedes, Culex* hits	Interpro results/comments
**Peptide hormones**				
Insulin like peptides (ILP)	GAWM01014579	m.5727	AAEL003000-RA, CPIJ001698-RA	Complete; ILP domain plus signal peptide
	GAWM01005128	m.24576	AAEL000973-RA, CPIJ018049-RA	Complete; ILP domain plus signal peptide
	GAWM01016117	m.6392	n/a	Complete; ILP domain plus signal peptide
**Nuclear receptors**				
Ecdysteroid receptor (EcR)	GAWM01005833	m.26438	AAEL009600-RA, CPIJ002963-RA	Complete; Ecdysteroid receptor family, zinc finger nuclear hormone receptor domain
	GAWM01005835	m.26440	AAEL009600-RA, CPIJ002963-RA	Complete; Ecdysteroid receptor family, zinc finger nuclear hormone receptor domain
Ultraspiracle (USP)	GAWM01006331	m.27928	AAEL000395-RB, CPIJ016024-RA	Complete; Retinoid X receptor/HNF4 family; zinc finger, nuclear hormone receptor domain
	GAWM01016442	m.6548	AAEL000395-RB, CPIJ016024-RA	Putative; zinc finger, nuclear hormone receptor domain
	GAWM01016447	m.6550	AAEL000395-RB, CPIJ016024-RA	Complete; Retinoid X receptor/HNF4 family; zinc finger, nuclear hormone receptor domain
**Target genes**				
E74A (ETS transcription factor)	GAWM01003891	m.20620	AAEL000741-RA, CPIJ003068-RA	Complete; ETS domain; Winged helix-turn-helix DNA-binding domain
E75B (nucelar receptor)	GAWM01003259	m.18897	AAEL007397-RA, CPIJ016641-RA	Complete; Zinc finger, nuclear hormone receptor domain
	GAWM01003260	m.18898	AAEL007397-RC, CPIJ007963-RA	Complete; Zinc finger, nuclear hormone receptor domain
	GAWM01001764	m.14388	AAEL007397-RC, CPIJ007963-RA	Partial (no zinc finger); Nuclear hormone receptor domain; steroid hormone receptor
Vitellogenin (Vg)	GAWM01013694	m.53805	AAEL006138-RA, CPIJ010190-RA	Partial (no VonWillebrand domain); signal 1-27, lipid transport protein domain (N terminal), beta sheet shell, superhelical region, disulfide bridges, vitellogenin open beta sheet.
	GAWM01006233	m.27656	AAEL006126-RA, CPIJ001357-RA	Partial (no signal peptide); lipid transport domain (N terminal), beta sheet shell, superhelical region, disulfide bridges, vitellogenin open beta sheet, VonWillebrand factor type D domain in C terminal, stop codon
Lipophorin (Lp), ApoLp-III	GAWM01018668	m.9284	AAEL008789-RA, CPIJ007775-RA	Complete; ApoLp-III domain, signal peptide
Vitellogenic carboxypeptidase (VCP)	GAWM01009232	m.36432	AAEL009291-RA, CPIJ008608-RA	Complete; signal peptide, serine carboxypeptidase
	GAWM01012606	m.48915	AAEL009291-RA, CPIJ008608-RA	Complete; signal peptide, serine carboxypeptidase
**Regulation**				
Seven-up (svp); COUP-TF	GAWM01004905	m.23485	AAEL006916-RA, CPIJ801727-RA	Complete; has COUP-TF DNA binding domain with two zinc fingers
**Target receptor**				
lipophorin/ldl receptor	GAWM01007791	m.32063	AAEL012251-RA, CPIJ018375-RA	Complete; LDL receptor class A/B repeats, EGF-like domain, TolB-like domain

**Table 2:** Transcriptome-level differential expression of vitellogenesis genes in blood-fed female C. sonorensis.

			Ten vs. BE	Ten vs. BL	BE vs. BL				
Description	Acc. no.	Seq. no.	Log2 change	P	Log2 change	P	Log2 change	P	notes
Insulin-like peptide	GAWM01014579	m.5727	−2.47	0.02	0.2	0.7	2.7	0.009	downregulated, then late blood induced
	GAWM01005128	m.24576	0.62	0.13	0.08	0.83	−0.54	0.18	not DE with blood feeding
	GAWM01016117	m.6392	−1.5	0.006	1.23	<0.0001	2.72	<0.0001	downregulated, then late blood induced
Ecdysteroid receptor	GAWM01005833	m.26438	0.34	0.38	−0.06	0.88	−0.4	0.31	not DE with blood feeding
	GAWM01005835	m.26440	0.19	0.66	−0.22	0.56	−0.41	0.34	not DE with blood feeding
Ultraspiracle receptor	GAWM01006331	m.27928	1.3	<0.0001	−0.03	0.92	−1.33	<0.0001	early blood induced
	GAWM01016442	m.6548	0.95	0.016	0.32	0.4	−0.64	0.09	not DE with blood feeding
	GAWM01016447	m.6550	2.03	<0.0001	0.08	0.854	−1.95	<0.0001	early blood induced
E74A	GAWM01003891	m.20620	2.13	<0.0001	1.02	0.01	−1.11	0.002	early blood induced
E75B	GAWM01003259	m.18897	−1.86	0.05	0.62	0.3	2.48	0.008	downregulated, then late blood induced
	GAWM01003260	m.18898	7.05	0.0016	5.17	0.02	−1.88	0.03	early and late blood induced
	GAWM01001764	m.14388	−1.84	<0.0001	−1.6	<0.0001	0.22	0.61	not DE with blood (high teneral)
Vitellogenin	GAWM01006233	m.27656	8.03	0	3.16	0	−4.87	0	blood induced, early>late
	GAWM01013694	m.53805	3.77	0	>14.04	0	>10.27	0	Blood induced, late>early
Apolipophorin-III	GAWM01018668	m.9284	−1.81	<0.0001	−0.94	0.004	0.88	0.04	downregulated with blood feeding
Vg carboxypeptidase	GAWM01009232	m.36432	−0.168	0.65	0.9	0.004	1.08	0.0023	late blood induced (low)
	GAWM01012606	m.48915	0.097	0.74	1.9	<0.0001	1.78	<0.0001	late blood induced (low)
Seven-up	GAWM01004902	m.23478	−4.46	6.37E-09	−1.1959	0.003	3.26	<0.0001	down with blood feeding, but increases late
	GAWM01004904	m.23481	−5.06	2.77E-06	−0.9221	0.07	4.13	0.0001	down with blood feeding, but increases late
	GAWM01004905	m.23485	−4.2141	0.0003	−1.39	0.0277	2.824	0.0163	down with blood feeding, but increases late
Lipophorin receptor	GAWM01007791	m.32063	−3.0201	4.74E-12	−1.848	1.87E-08	1.1721	0.0072	down with blood feeding, but increases late
	GAWM01007792	m.32066	−2.5085	1.51E-10	−2.1162	2.02E-10	0.3923	0.3168	downregulated with blood feeding

Ten (teneral), BE (early blood-fed), BL (late blood-fed) transcriptomes.

### Differential expression analysis revealed useful reference or “housekeeping” genes for use in future studies of *C. sonorensis* gene expression

A well annotated transcriptome and differential expression analysis provides a valuable tool for follow up experiments and validation of gene expression with quantitative reverse-transcriptase PCR (qRT-PCR). Likewise, ongoing studies in our laboratory are aimed at determining the temporospatial expression of key genes identified in this study. Of great need and value to these studies is the availability of a reliable and truly non-differentially expressed housekeeping gene, or reference gene. It is conventional to use ribosomal protein genes and other structural proteins such as tubulin and actin as reference genes for calibrating expression of targets in qRTPCR. However, in many instances these genes are differentially expressed across treatment groups. Our differential expression analysis allowed us to examine the regulation of several ribosomal protein genes commonly used as reference genes for qRTPCR including *rpS18, rpL32, rpS7*. All three of these genes were differentially expressed across at least one of the feeding states (P≤0.01; Table S12 in File S1). In addition, we identified a number of genes that were not differentially expressed for putative use as reference genes (e.g. *rpS12, rpS3a, rpL37*, etc.); however, their expression values were too low (i.e., transcript abundance <30 FPKM) and when tested in qRTPCR assays, their amplicon abundance did not cross the threshold cycle (C_T_) within the standard 40 cycles (data not shown). At least one gene in our transcriptome, coding for the protein elongation actor 1-beta (*EF1b*; Acc. no. GAWM01010754), was not differentially expressed across any of the conditions, and, importantly, was highly expressed (FPKM>100). Preliminary analysis has shown that this reference gene works reliably in qRTPCR analysis, yielding reproducible C_T_ values not only in whole midges but also in a variety of tissues from salivary gland to midgut (Nayduch et al., in preparation).

## Conclusions

Unlike many other important hematophagous insect vectors, the genome and transcriptome(s) of *C. sonorensis* have not been available as a resource for *Culicoides* vector biologists, which has hindered genetic and functional genomics studies as well as detailed understanding of molecular, cellular and physiological processes of this important vector. The adult female reference transcriptome and differential expression analysis presented here represents a critical milestone and fills a profound gap in *C. sonorensis* biology such as understanding the genetic basis of anautogeny, hematophagy and other key physiological processes specific to the midge, and is paramount for the development of novel approaches to vector control. Moreover, these resources will serve as the framework for follow up studies that will include virus challenged midges to unravel midge vector competence as well as a rich dataset for the assembly and annotation of the *C. sonorensis* genome, which is an ongoing project of the *Culicoides* Genome and Transcriptome Alliance (CGAT). CGAT is an international partnership between several institutions including the Agricultural Research Service of the United States Department of Agriculture (USDA-ARS), Clemson University Genomics Institute (CUGI), The Pirbright Institute, and the European Bioinformatics Institute (part of the European Molecular Biology Laboratory; EBI-EMBL). The aim is to build more molecular genetic resources including several transcriptomes and a genome for this important vector.

## Supporting Information

Figure S1 **Transcriptional response to sucrose feeding in female** ***Culicoides sonorensis*** **.** Numbers of genes classified within GO-slim categories that were upregulated (blue) or downregulated (red) in response to sucrose feeding are shown, including comparisons of (A) teneral versus an early sucrose meal (B) teneral versus an late sucrose meal, and (C) early sucrose meal versus late sucrose meal.
(TIF)

Figure S2 **Multiple alignment of midge and mosquito vitellogenins.** Full amino acid sequences for *Culicoides sonorensis, Aedes aegypti* and *Culex pipiens* vitellogenins were aligned using ClustalW algorithm in the Mega5 software (www.megasoftware.net).
(PPTX)

File S1 **Tables S1–S12.** Table S1. Sample trimming results of 2×101 bp PE Illumina. Table S2. BlastX alignment of the *Culicoides* unigene to the NR, and *Aedes* and *Culex* transcriptomes. Table S3. Unique GO accessions of the *Culicoides* transcriptome mapped to the GO database. Table S4. Top 100 genes with at least 2-fold increase in abundance in early response to a blood meal. Table S5. Effect of blood feeding on differential expression of genes in female *Culicoides sonorensis*. Table S6. Significant differentially expressed genes between teneral and early blood-fed *C. sonorensis*. Table S7. Significant differentially expressed genes between teneral and late blood-fed *C. sonorensis*. Table S8. Significant differentially expressed genes between early and late blood-fed *C. sonorensis*. Table S9. Significant differentially expressed genes between teneral and early sugar-fed *C. sonorensis*. Table S10. Significant differentially expressed genes between teneral and late sugar-fed *C. sonorensis*. Table S11. Effect of sucrose feeding on differential expression of genes in female *Culicoides sonorensis*. Table S12. Differential expression of putative housekeeping or reference genes in *C. sonorensis*.
(XLSX)
