# Supplementary figures and images for: The Reference Transcriptome of the Adult Female Biting Midge (Culicoides sonorensis) and Differential Gene Expression Profiling during Teneral, Blood, and Sucrose Feeding Conditions
Source: PLoS One. 2014 May 27;9(5):e98123. doi: 10.1371/journal.pone.0098123 (PMC4035326; doi:10.1371/journal.pone.0098123)

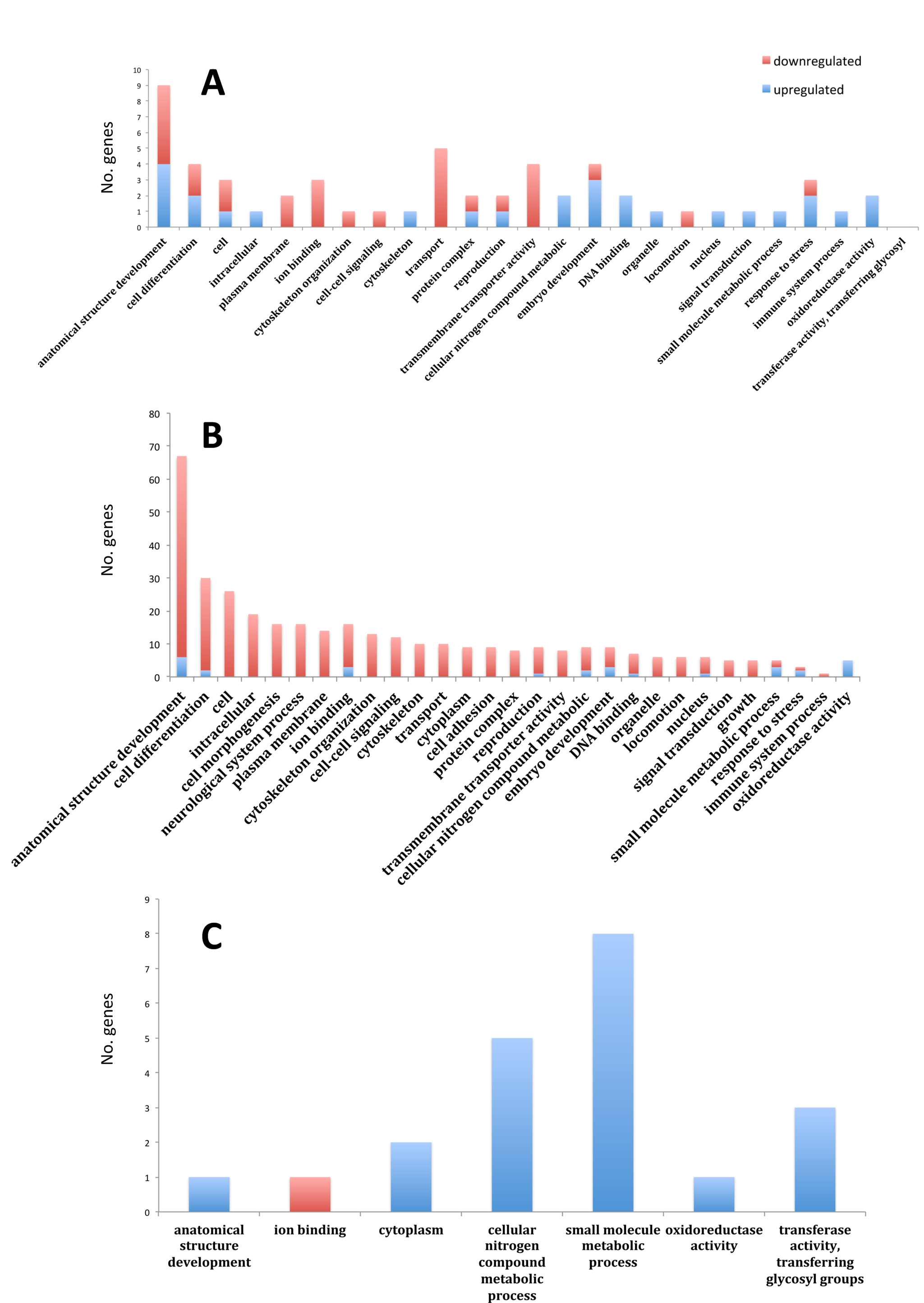

Supplement: Figure S1 — Transcriptional response to sucrose feeding in female Culicoides sonorensis . Numbers of genes classified within GO-slim categories that were upregulated (blue) or downregulated (red) in response to sucrose feeding are shown, including comparisons of (A) teneral versus an early sucrose meal (B) teneral versus an late sucrose meal, and (C) early sucrose meal versus late sucrose meal. (TIF) [file pone.0098123.s001.tif]

## Slide 1
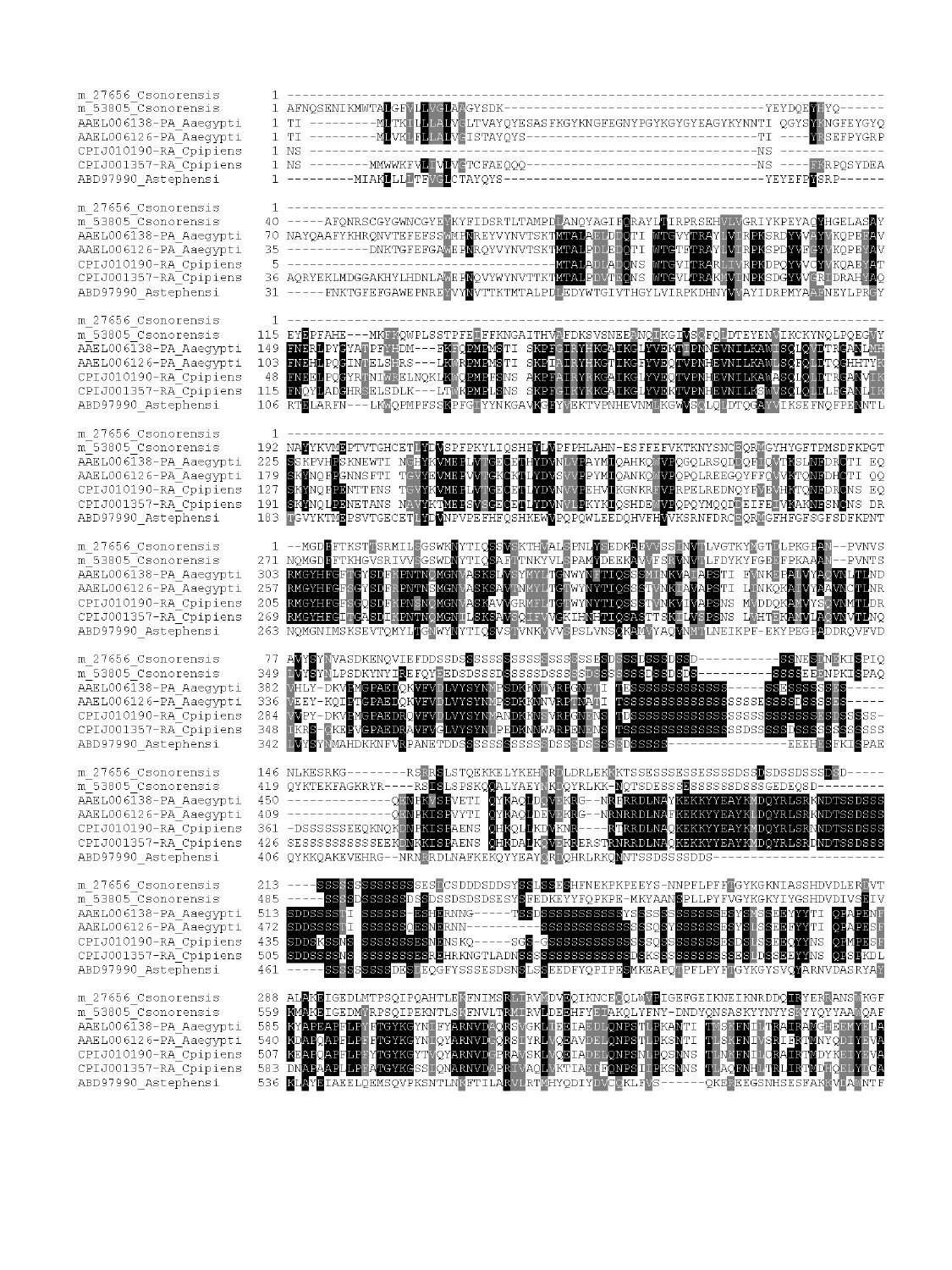

## Slide 2
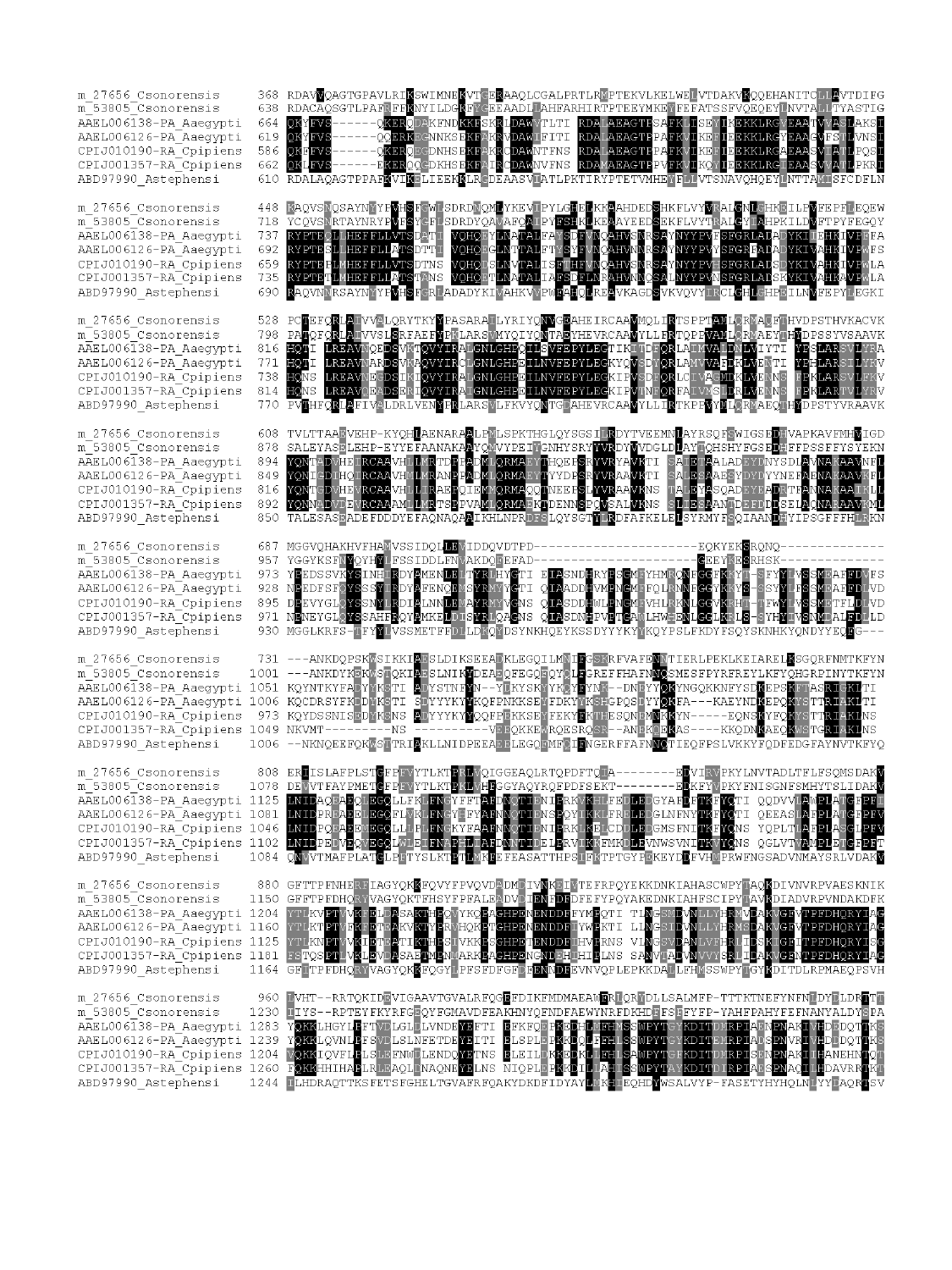

## Slide 3
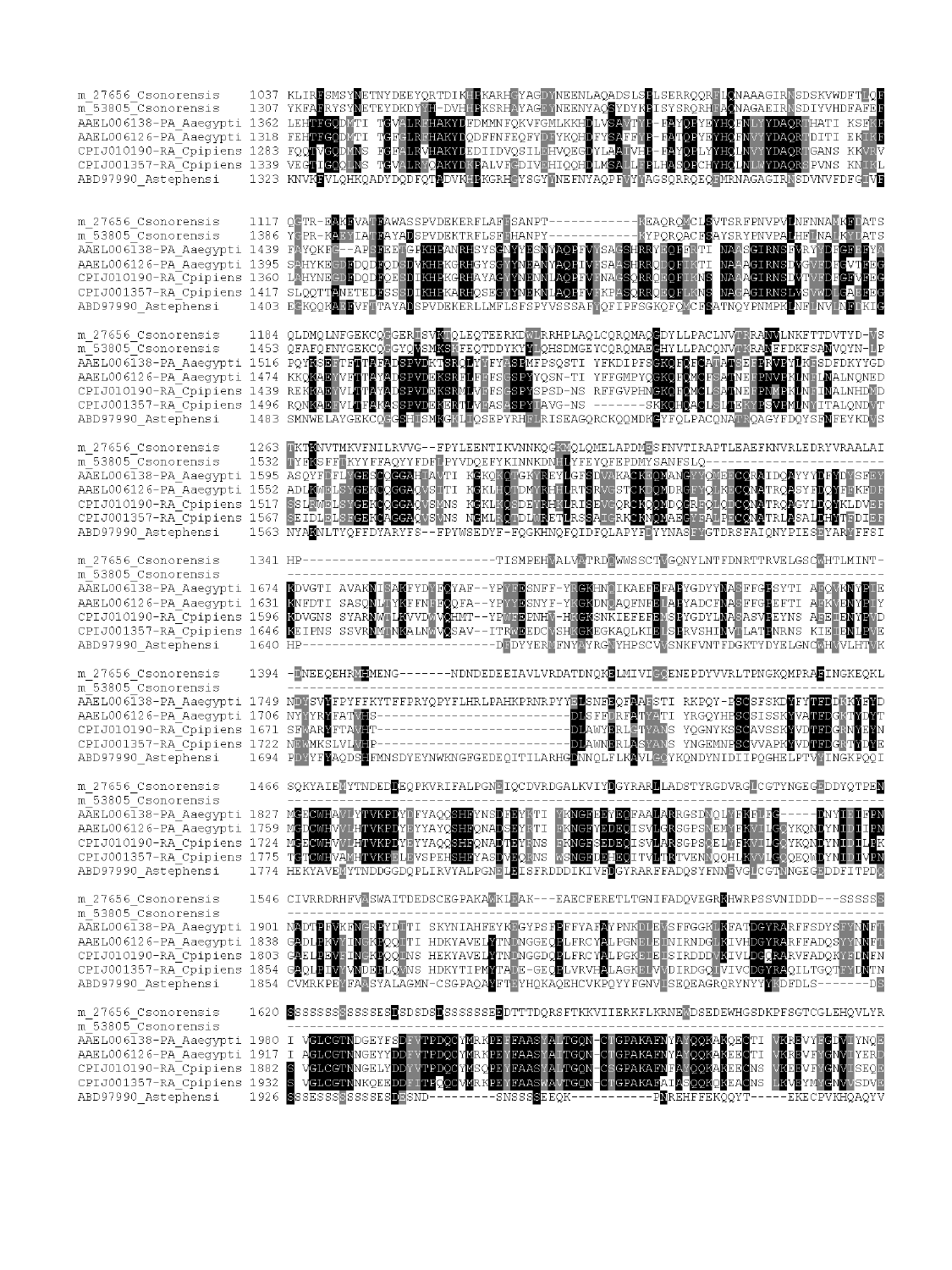

## Slide 4
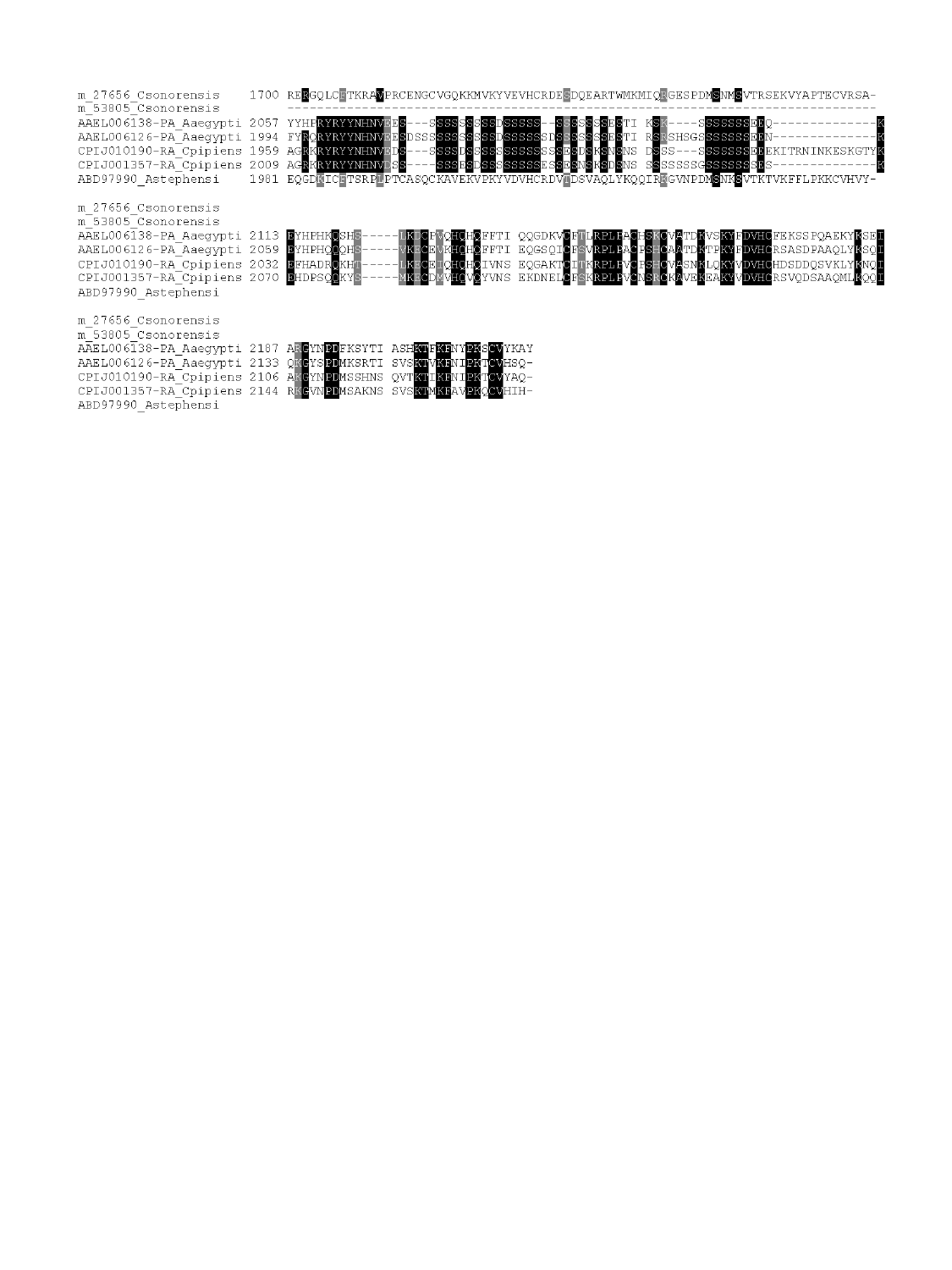

Supplement: Figure S2 — Multiple alignment of midge and mosquito vitellogenins. Full amino acid sequences for Culicoides sonorensis, Aedes aegypti and Culex pipiens vitellogenins were aligned using ClustalW algorithm in the Mega5 software (www.megasoftware.net). (PPTX) [file pone.0098123.s002.pptx]
